# Genomic Sequence of a *Megrivirus* Strain Identified in Laying Hens in Brazil

**DOI:** 10.1128/MRA.01438-18

**Published:** 2019-01-24

**Authors:** Priscilla F. Gerber, Huigang Shen, Ying Zheng, Ganwu Li, Zélia I. P. Lobato, Tanja Opriessnig

**Affiliations:** a Animal Science, School of Environmental and Rural Science, University of New England, Armidale, Australia; b Department of Veterinary Diagnostic and Production Animal Medicine, College of Veterinary Medicine, Iowa State University, Ames, Iowa, USA; c Laboratório de Pesquisa em Virologia Animal, Departamento de Medicina Veterinária Preventiva, Escola de Veterinária, Universidade Federal de Minas Gerais, Belo Horizonte, Minas Gerais, Brazil; d The Roslin Institute and The Royal (Dick) School of Veterinary Studies, University of Edinburgh, Midlothian, United Kingdom; KU Leuven

## Abstract

A new strain of chicken megrivirus was identified in fecal samples of layer chickens in a commercial flock in Minas Gerais, Brazil. It is most closely related to the family *Picornaviridae*, genus *Megrivirus*, species *Melegrivirus A*, and has an overall nucleotide identity of up to 85.1% with other megrivirus strains.

## ANNOUNCEMENT

The *Picornaviridae* family currently consists of 40 genera (1), with at least 13 of these genera identified from avian sources (2). Avian picornaviruses of the genera *Megrivirus*, *Gallivirus*, and *Avisivirus* have been frequently identified in healthy and diseased poultry (3–5). Recent metagenomics studies focusing on the poultry gut virome have identified an ever-growing number of enteric picornaviruses in fecal samples from broiler chickens (3–8). In this study, two pooled fecal samples (2 g of fresh fecal material from five sites each in two sheds) were collected from a 102-week-old and a 57-week-old commercial laying flock in Minas Gerais State, Southeast Brazil, in August 2012. No health problems had been reported in the examined flocks.

Total nucleic acid was extracted from a 1:5 dilution of fecal samples using the MagMAX pathogen RNA/DNA kit (Thermo Fisher Scientific, MA) with KingFisher (Thermo Fisher Scientific) (9). Double-stranded cDNA was synthesized using the NEXTflex rapid transcriptome sequencing (RNA-seq) kit (Bioo Scientific Corp., TX). The sequencing library was prepared using the Nextera XT DNA library preparation kit (Illumina, CA) with dual indexing. The pooled libraries were sequenced on an Illumina MiSeq platform at the next-generation sequencing (NGS) lab located in the Veterinary Diagnostic Laboratory at Iowa State University, using the 300-cycle v2 reagent kit (Illumina) to generate 150 base-pair paired-end reads by following standard Illumina protocols. Raw reads of each sample were demultiplexed automatically on the MiSeq platform with the default settings.

Raw sequencing reads were preprocessed using Trimmomatic v0.36 to remove adapters and trim low-quality ends (10). Raw reads and preprocessed reads were subjected to sequencing quality analysis with FastQC (https://www.bioinformatics.babraham.ac.uk/projects/fastqc/) to ensure the efficiency of cleaning. Cleaned reads were fed to a comprehensive reference-assisted virus genome assembly pipeline (9, 11) with modifications. Briefly, the cleaned reads were aligned to the host reference genome using the Burrows-Wheeler Aligner MEM algorithm (BWA-MEM) (12); the nonhost reads were classified using Kraken v1.0 (13), and the unclassified reads were classified using Kaiju v1.6.2 (14); KronaTools v2.7 (15) was used to generate hierarchical classification results in which chicken picornavirus was identified; additional (supplementary) reads were collected before *de novo* assembly (9) by mapping the quality-trimmed reads to publicly available chicken picornavirus genomes using BWA-MEM (12), SAMtools (16), and seqtk (https://github.com/lh3/seqtk); contigs were assembled using Assembly by Short Sequences (ABySS) v1.3.9 (17); and the resulting contigs were manually curated to remove contaminated (nonviral) contigs and to trim chimeric (misassembled) contigs in the SeqMan Pro DNASTAR Lasergene 11 core suite.

Eight contigs of chicken picornaviruses were assembled from the raw data (SRA accession no. SRR8290010) with an *N*_50_ value of 1,268 bp, and specific primers (provided upon request) were then designed to perform reverse transcriptase PCR (RT-PCR) and close the gaps by sequencing of the Nextera XT DNA library of the amplicon on a MiSeq platform (SRA accession no. SRR8290011). Finally, a near-complete sequence of a megrivirus, chicken picornavirus MG/9567, was identified with a genome length of 9,567 nucleotides. The genome had 78.3% nucleotide similarity with the reference *Megrivirus C* isolate BL 21 (GenBank accession no. KF961186).

The chicken picornavirus MG/9567 genome shared the highest deduced polyprotein amino acid similarity (94.9%) with chicken picornavirus 5 isolate 27 (GenBank accession no. KF979336), which originated from a chicken sample collected in Hong Kong in 2008, and shared 87.7% amino acid (aa) identity with chicken megrivirus strain 3R, collected from a healthy chicken in Brazil in 2018 (GenBank accession no. MG846465). The genome nucleotide similarities were 85.2% and 78.3%, respectively.

### Data availability.

The genome sequence reported here has been deposited in GenBank under the accession no. MH806866. The sequence data are available under SRA accession no. SRR8290010 and SRR8290011.
